# Postoperative Stroke Following Anterior Cervical Spine Surgery: A Case Report

**DOI:** 10.7759/cureus.63846

**Published:** 2024-07-04

**Authors:** Andreea D Butnariu, Ioana Miron, Bogdan David, Dan M Visarion, Violeta I Pruna, Viorel M Pruna

**Affiliations:** 1 Neurosurgery Department, Bagdasar-Arseni Clinical Emergency Hospital, Bucharest, ROU; 2 Neurosurgery Department, Carol Davila University of Medicine and Pharmacy, Bucharest, ROU; 3 Ophthalmology Department, CF2 Clinical Hospital, Bucharest, ROU

**Keywords:** atherosclerosis, carotid artery atheromatosis, postoperative stroke, cervical disc herniation, mechanical thrombectomy, anterior cervical discectomy and fusion, stroke

## Abstract

Vascular complications succeeding anterior cervical spine surgery are rare, but their consequences represent a major burden for the patient. Cerebral infarction following anterior cervical discectomy and fusion (ACDF) is uncommon. However, screening for risk factors before surgery should become mandatory. We present the case of a patient with no significant medical history who underwent ACDF for a C5/C6 herniated disc with myelopathy. Although the surgery was uneventful, after the surgery, partial right palpebral ptosis and miosis were noted, suggestive of Horner syndrome. On the fifth postoperative day, the patient experienced left hemiplegia and drowsiness. An emergency CT scan and cerebral MRI revealed ischemia in the right middle cerebral artery territory. The patient was transferred to a neurology center for mechanical thrombectomy, which revealed a complete occlusion of the right internal carotid artery. The procedure had to be halted due to blood extravasation at the internal carotid artery bifurcation to prevent further complications. An angio-CT examination of the cervical arteries exposed a soft atheromatous plaque on the right internal carotid artery, immediately after the bifurcation. Despite the patient having no significant medical history, blood tests indicated dyslipidemia. At the two-month follow-up, the patient remained hemiplegic, with mild dysphasia. Performing carotid and vertebral Doppler ultrasound before cervical spine surgery might be useful, whenever possible, to assess high-risk factors for ischemic events and avoid such debilitating complications.

## Introduction

Due to the intrinsic anatomical particularities, cervical spine surgery carries the risk of vascular injury to the vertebral arteries or, in even rarer instances, the carotid arteries [1,2]. Anterior cervical discectomy and fusion (ACDF) is an important surgical option for treating cervical degenerative disease, with a low incidence of complications and good clinical results [3,4]. ACDF requires identifying the carotid artery followed by mild lateral retraction to expose the anterior surface of the cervical spine [5]. Although prolonged retraction may alter the hemodynamics of the carotid artery and cause serious complications in atherosclerotic patients [6], carotid artery injury and stroke secondary to prolonged retraction remain extremely rare complications in ACDF [7]. Multiple studies have shown that carotid artery manipulation during this surgical approach may alter the normal blood flow, significantly reducing the vessel’s cross-sectional area [8,9]. Additionally, the dislodgment of atherosclerotic plaques during manipulation of the carotid artery can be a potential risk factor for intracranial embolus and stroke. Screening for risk factors and intermittent release of the retractors in case of prolonged surgeries might help in preventing this major complication [9].

## Case presentation

A 55-year-old female with no medical history other than class II obesity presented with bilateral upper limb weakness, cervico-brachialgia, and paresthesia, more prominent on the left side, and was diagnosed with Frankel D cervical spinal cord compression syndrome at the C5 level. The patient was not on any chronic medication.

**Figure 1:**
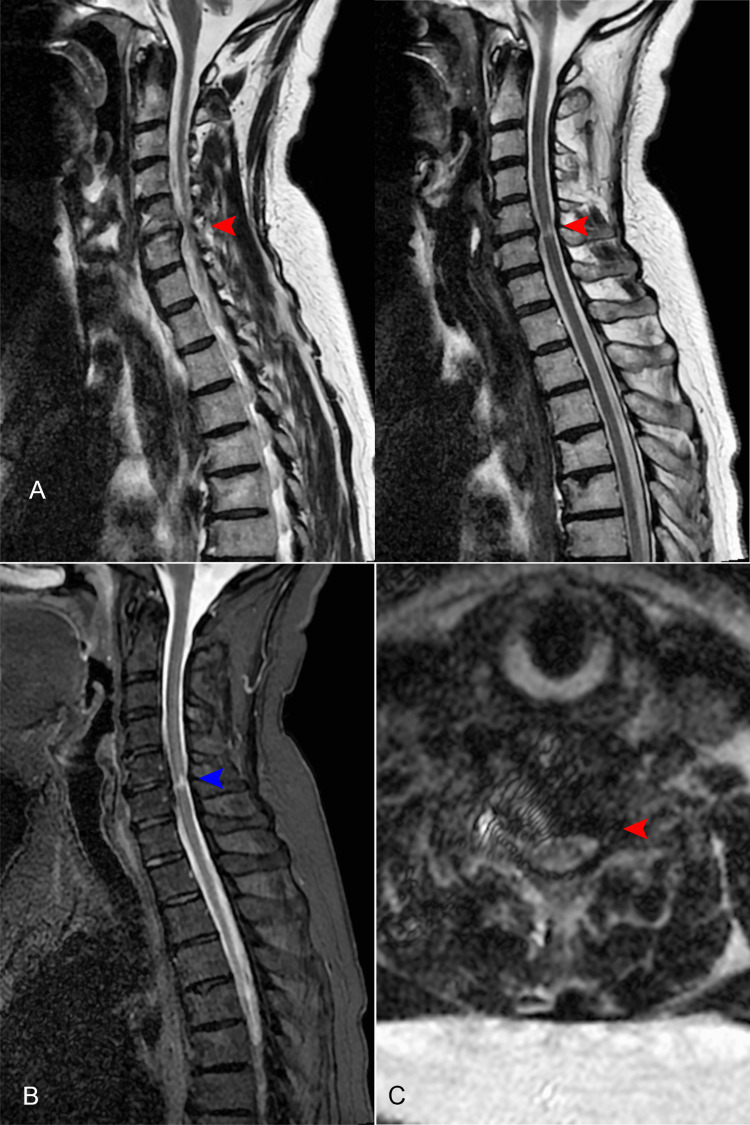
Preoperative MRI showing cervical disc herniation at the C5-C6 level compressing the spinal cord (red arrows) and signs of cervical myelopathy (blue arrow) due to disc compression on the spinal cord. (A) T2 sagittal section; (B) short tau inversion recovery sagittal section; (C) T2 axial section.

The cervical MRI showed anterior osteophytes at the C4-C6 level, and a C5 disc herniation compressing the spinal cord and the C6 root bilaterally (Figures 1A, 1C). Sagittal short tau inversion recovery sequences were highly suggestive of compressive myelopathy, with an intramedullary hyperintense area next to the herniated disc (Figure 1B).

**Figure 2:**
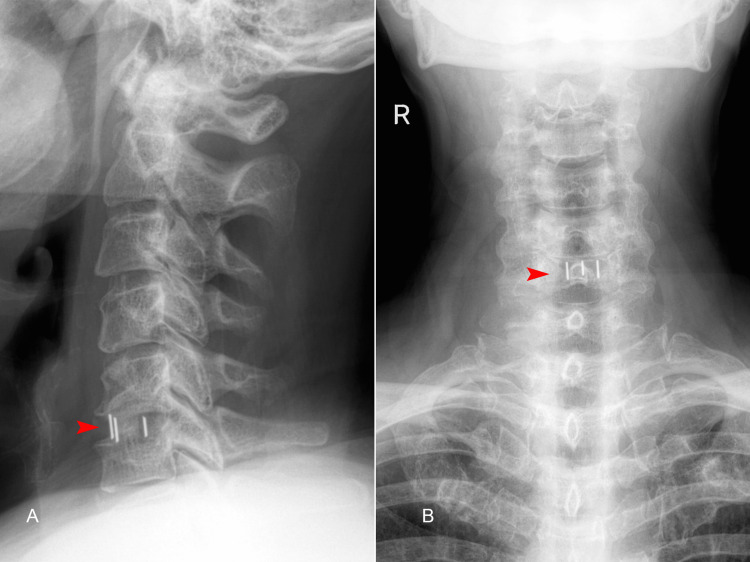
Postoperative X-ray of the cervical spine (A) lateral and (B) anteroposterior views, highlighting the interbody cage at the C5/C6 level (red arrows).

The electromyography revealed bilateral C6 radicular compression with moderately chronic denervation activity. The patient underwent C5-C6 ACDF using a cervical interbody cage and synthetic bone paste. We opted for a right anterior cervical approach using self-retaining retractors for exposure. The procedure lasted two hours, with 70 minutes of retraction and a blood loss of 30 mL. During surgery, the patient experienced a mild blood pressure decrease, with blood pressure dropping to 100/60 mmHg, which was promptly corrected with rapid crystalloid infusion. Blood pressure was maintained around 120/80 mmHg until the end of surgery. Postoperatively, the patient recovered well, with complete remission of motor deficits. However, mild anisocoria (right eye miosis) and mild right palpebral ptosis were noted immediately after surgery. The following day, the patient was mobilized with a cervical collar. Thromboprophylaxis with low-molecular-weight heparin (LMWH) was administered after the surgery daily. A postoperative cervical radiograph performed 24 hours after surgery confirmed the correctly positioned graft (Figure 2).

**Figure 3:**
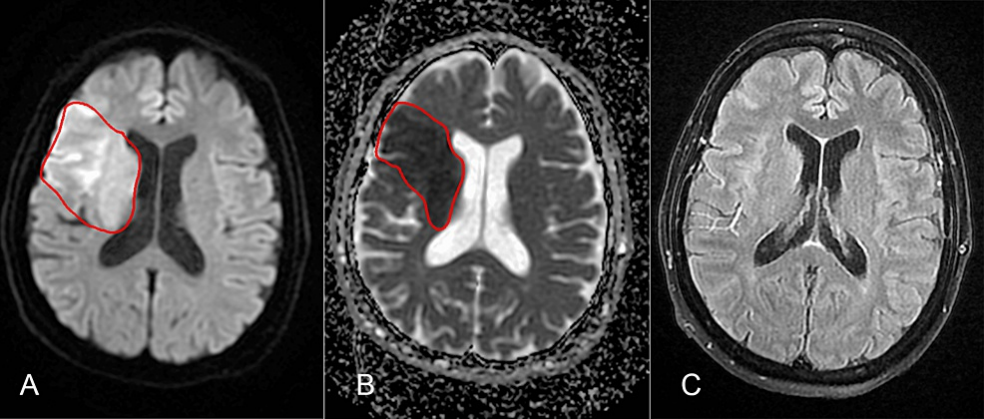
Cerebral MRI performed shortly after the stroke onset. (A) Diffusion-weighted imaging sequence; (B) apparent diffusion coefficient sequence; (C) fluid-attenuated inversion recovery sequence reveals a recent ischemic stroke in the territory of the right middle cerebral artery.

On the fifth postoperative day, the patient reported intense headache and dizziness while waking up, presenting drowsiness and left hemiplegia. An emergency brain CT scan was normal, without signs of hemorrhage or hypodense lesions (Alberta stroke program early CT score 10). The most likely diagnosis was an acute stroke and the patient received 300 mg aspirin and 80 mg statin. A specialized neurology center proficient in thrombolysis and thrombectomy was promptly contacted. As the exact onset of symptoms was unknown (wake-up stroke), a cerebral diffusion-weighted imaging (DWI)/fluid-attenuated inversion recovery (FLAIR) MRI (DWI-FLAIR mismatch would suggest an acute stroke in the first four to five hours) was requested. The MRI revealed recent thrombosis of the right middle cerebral artery (Figure 3). Upon obtaining the MRI results, we received approval to transfer the patient for thrombectomy. At the time of transfer, the patient had a Glasgow Coma Scale score of 13 with left hemiplegia. The elapsed time from the identification of the wake-up stroke to the initiation of treatment was four hours.

**Figure 4:**
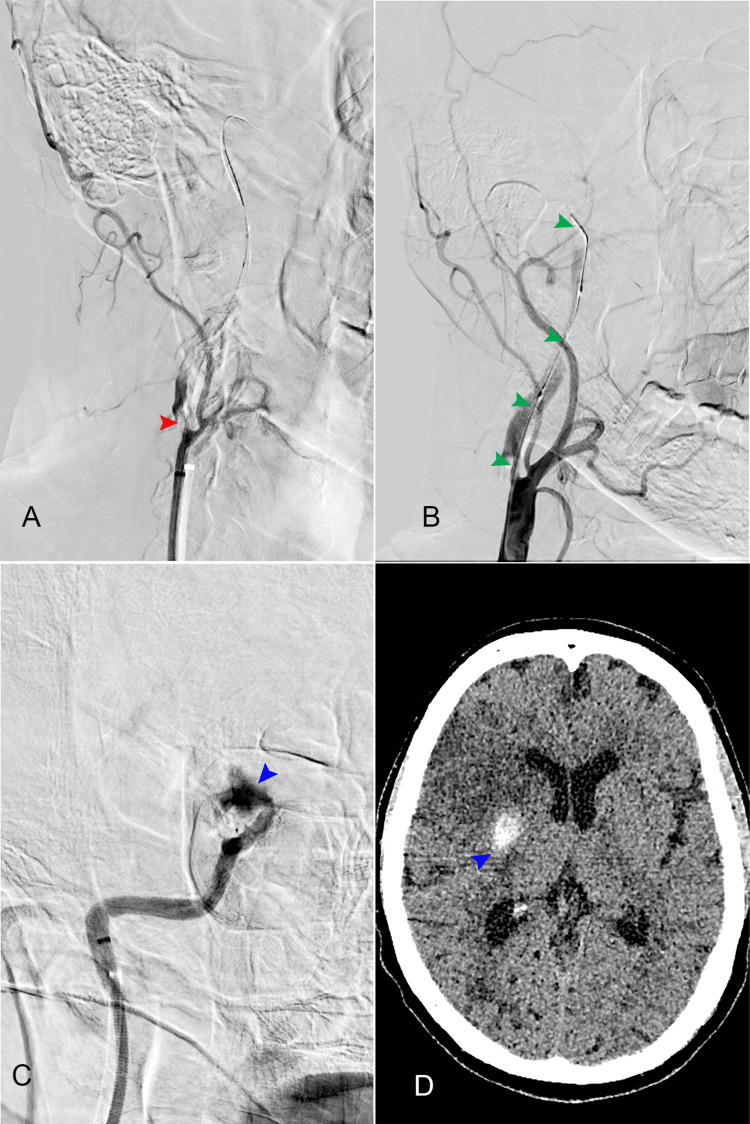
Angiography images from the thrombectomy procedure. (A) Right carotid artery bifurcation and occlusion of the internal carotid artery; red arrow: the obstruction near the bifurcation; (B) green arrows: partial visualization of the right internal carotid artery in the proximal segments; (C) blue arrow: blood extravasation during the procedure at the carotid T level; (D) CT scan performed 24 hours after the procedure, depicting intracerebral hemorrhage (blue arrow).

**Figure 5:**
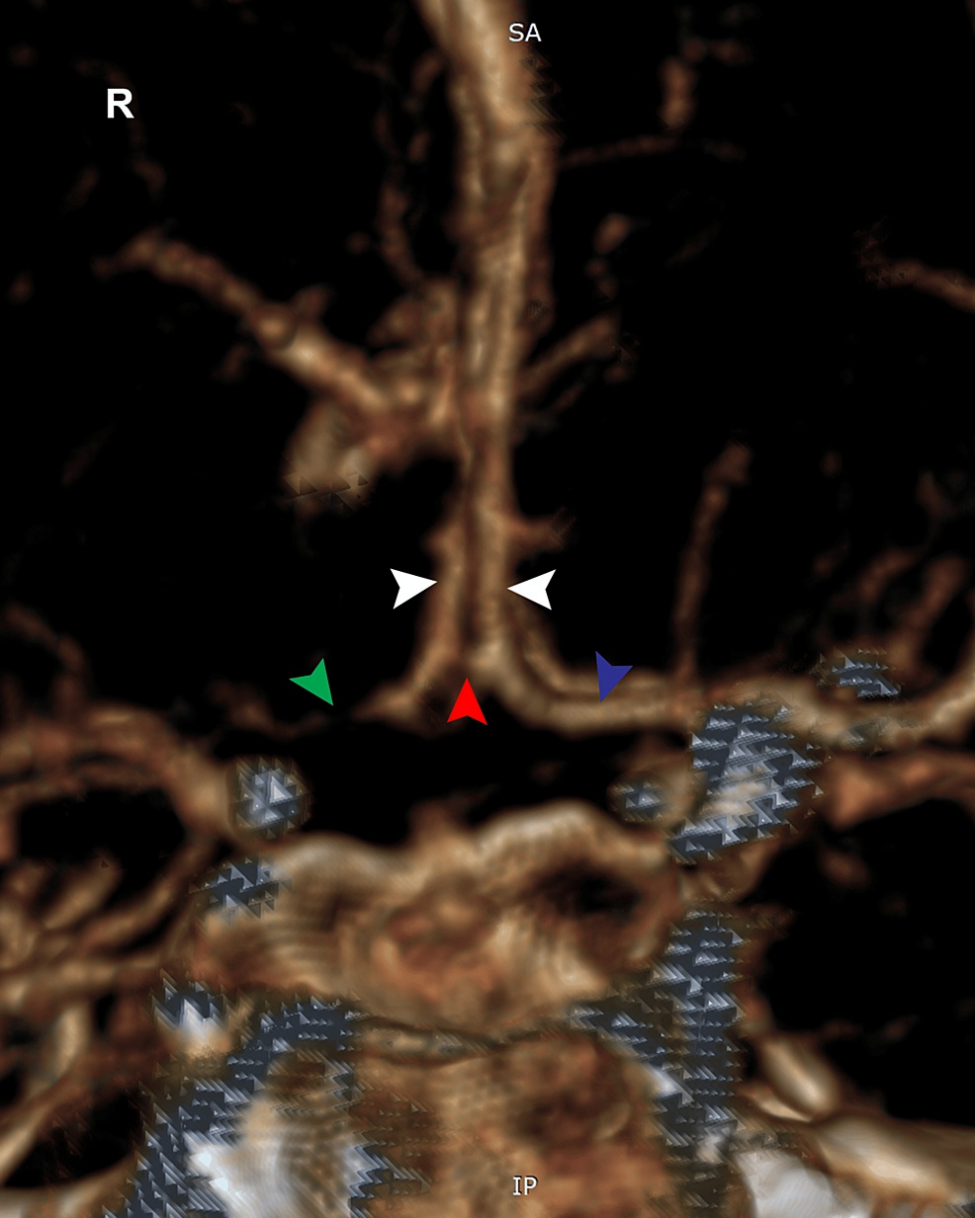
Cerebral angiography of the left internal carotid artery demonstrates the patency of the anterior communicating artery (ACoA) complex. The bilateral A1 segments are indicated by white arrows, the ACoA is marked by a red arrow, and the occlusion of the right A1 segment is highlighted by a green arrow. The blue arrow indicates the permeability of the left A1 artery.

Catheterization of the left femoral artery was performed, followed by selective injection of contrast into the right common carotid artery, revealing occlusion of the right internal carotid artery at the origin (Figure 4, Panels A, B) and the patency of the anterior communicating artery complex (Figure 5). Balloon angioplasty of the right internal carotid artery was conducted. During the procedure, contrast extravasation was observed at the bifurcation of the internal carotid artery, prompting the decision to maintain balloon inflation (Figure 4, Panel C). After the angioplasty, a partial thrombus was extracted from the right internal carotid artery. Recognition of contrast extravasation, indicative of bleeding, led to the termination of the procedure to mitigate further risks to the patient. Based on these findings, it is highly probable that the patient experienced a tandem occlusion, including stenosis with occlusion of the cervical internal carotid artery at the bifurcation and a thromboembolic occlusion of the middle cerebral artery.

Twenty-four hours after the attempted procedure, a brain CT scan was performed (Figure 4, Panel D), revealing right frontal ischemia and a right parietal intergyral hemorrhage, along with hemorrhage in the interpeduncular cistern. The cerebral CT scan performed 72 hours later demonstrated right frontoparietal ischemia with remission of the hemorrhagic area.

**Figure 6:**
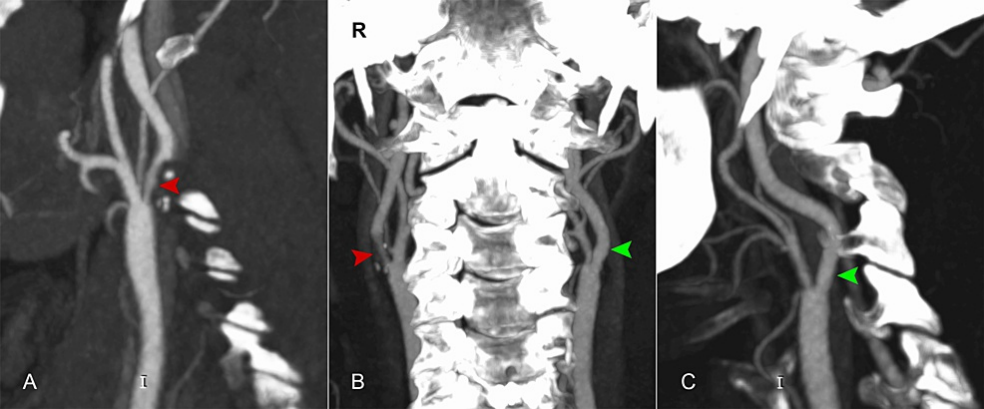
The left common carotid artery and left internal carotid artery are permeable with normal caliber (green arrows); the right internal carotid artery: soft plaque at the origin, causing 50% stenosis (red arrows). (A) Right sagittal section; (B) coronal section; (C) left sagittal section.

While hospitalized in the neurology department, a brain angio-CT showed a soft atherosclerotic plaque, resulting in a 50% narrowing of the right internal carotid artery, confirming the suspicion that the ischemic stroke was likely caused by atheromatous occlusion of the internal carotid artery (Figure 6).

Additionally, blood tests indicated the presence of dyslipidemia: low-density lipoprotein, 163 mg/dL (0-100 mg/dL); triglycerides, 312 mg/dL (0-150 mg/dL); and total cholesterol, 272 mg/dL (0-200 mg/dL). On discharge, the patient was transferred to a neuromotor recovery unit with left hemiplegia, partial right palpebral ptosis, and mild dysarthria.

**Figure 7:**
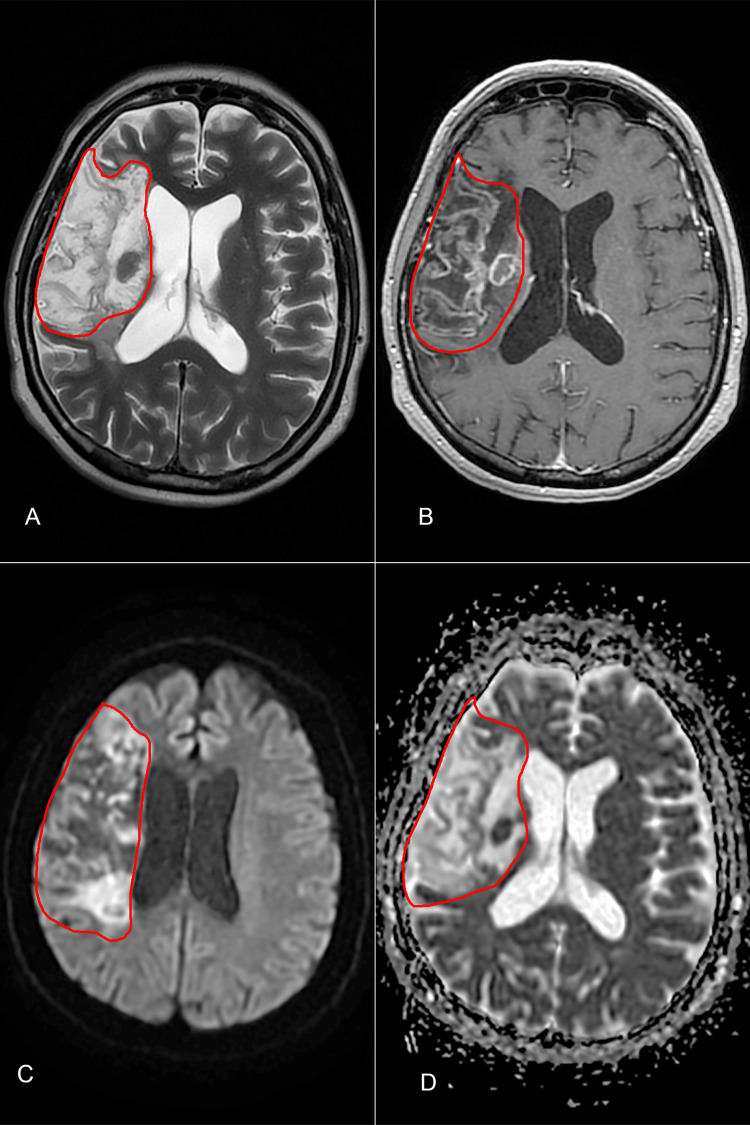
Cerebral MRI at the two-month follow-up depicting ischemic sequelae in the right frontoparietal region, with the area outlined in red. (A) T2 sequence; (B) T1 + C sequence; (C) diffusion-weighted imaging sequence; (D) apparent diffusion coefficient sequence.

At the two-month neurosurgical follow-up, the patient remained hemiplegic, with a modified Rankin score of 4, recovering from dysarthria, and exhibiting only mild motor function in the upper limb. A cerebral MRI was performed, showing ischemic sequelae in the right frontoparietal region (Figure 7).

## Discussion

Perioperative internal carotid artery stroke is a very rare complication after cervical spine surgery, but the clinical consequences of such an ischemic event represent a major concern [2]. The incidence is extremely low, with Hartl et al. reporting a rate of 1:17,625 in their extensive study [4]. Proper exposure of the anterior cervical plane necessitates retraction; however, maintaining the retractors in position for long periods, especially in patients with atherosclerotic carotid arteries, can increase the risk of ischemic events [10,11]. Under these conditions, soft plaques can be mobilized during retraction [2]. Blood stasis can further lead to thrombosis, particularly in the presence of atheromatosis or stenosis [12], although there have also been cases of fatal strokes in patients with normal carotid arteries [7]. Ischemic events related to the carotid artery can occur due to cerebral hypoperfusion resulting from intraoperative hypotension [2,10,11], but hypotension alone is not enough to produce an ischemic stroke [13]. Pollard et al. measured the carotid artery flow in 15 patients undergoing anterior cervical spine surgeries and found that during retraction, the cross-sectional area decreased first by 14%, leading to a 30% decrease by the end of the procedure, and in one case, the decrease was more than 67%. The authors stressed that retraction must be used with caution, and in the occurrence of intraoperative hypotension, it might be prudent to temporarily release retraction until the blood pressure normalizes [6]. Nevertheless, an ischemic event occurring five days after the surgery was most likely due to the patient’s risk factors, rather than a consequence of surgical manipulation of the carotid artery.

Our patient did not have any significant medical history and had never undergone a carotid and vertebral Doppler ultrasound. A lipid blood profile performed in the neurology unit demonstrated dyslipidemia, and the angio-CT of the cervical arteries revealed an atheromatous plaque at the origin of the right internal carotid artery. Unfortunately, the preoperative blood test did not include a lipid blood profile by default, but we consider it important, especially in high-risk patients.

The surgery lasted two hours, with 70 minutes of retraction, and was uneventful, with minimal blood loss and minimal need of bipolar coagulation. However, mild right palpebral ptosis and right eye miosis were noted immediately after the surgery, raising suspicion of Horner’s syndrome. This complication occurs in 0.6% of cases undergoing ACDF, and most patients recover within the first six months [14]. It occurs most frequently at lower cervical levels, where the sympathetic trunk is closer to the medial border of the longus colli muscle [15].

Once we confirmed the acute ischemic stroke in the right middle cerebral artery, the two available options were intravenous thrombolysis (IVT) or mechanical thrombectomy. According to our national protocol, IVT is contraindicated within three months following cranial or spinal surgery, while more recent international guidelines restrict the procedure to 14 days after major surgery [16]. However, Voelkel et al. stated that IVT can be administered off-label if the risk-benefit ratio is in the patient’s favor, considering the bleeding risk of patients who underwent surgery shortly before the ischemic event [17]. For this subset of patients, with perioperative stroke (defined as an acute stroke occurring during surgery or within the first month after), mechanical thrombectomy can be lifesaving. However, results seem to be less favorable compared to patients with acute stroke without a recent surgical history [18]. In our case, the procedure was stopped due to blood extravasation at the internal carotid artery bifurcation.

The main risk factors for perioperative stroke in general are age over 65 years, carotid atherosclerosis, male sex, atrial fibrillation and hypertension, and intraoperative hypotension [10,13]. Mpody et al. studied the timing of postoperative stroke and found that most ischemic events occur within the first seven days after the surgery (defined as early stroke) and that mortality in these cases is higher compared to patients suffering from delayed postoperative stroke [19]. Moreover, patients diagnosed with carotid atherosclerosis who undergo ACDF have a statistically higher risk of experiencing a postoperative stroke [20]. Even though the patient did not have a relevant medical history other than class II obesity, untreated dyslipidemia can lead to atherosclerotic plaques. We consider it important to screen patients before surgery with Doppler ultrasound of the cervical vessels to identify potential carotid artery stenosis or atheromatous plaques that might be dislodged during retraction.

## Conclusions

Cerebral infarction is a rare postoperative complication following ACDF and screening for risk factors before surgery should become mandatory. Especially in atherosclerotic patients, prolonged retraction and intraoperative hypotension might cause an ischemic stroke. In early postoperative strokes where IVT is contraindicated, mechanical thrombectomy remains a possible therapeutic option. To prevent this vascular complication, in non-urgent cases of spine surgery, patients at risk for ischemic stroke should undergo carotid and vertebral Doppler ultrasound before surgery.
